# Micronutrient regulation of the DNA methylome

**DOI:** 10.3389/freae.2024.1409355

**Published:** 2024-04-26

**Authors:** Tiffany Leesang, Peter Lyon, Joey Pinzone, Luisa Cimmino

**Affiliations:** ^1^ Department of Biochemistry and Molecular Biology, Miller School of Medicine, University of Miami, Miami, FL, United States; ^2^ Sylvester Comprehensive Cancer Center, Miller School of Medicine, University of Miami, Miami, FL, United States

**Keywords:** DNA methylation, Ten-eleven translocation (TET) enzymes, 5-hydroxymethylation, vitamin A, vitamin B, vitamin C

## Abstract

The formation, inheritance, and removal of DNA methylation in the genome of mammalian cells is directly regulated by two families of enzymes–DNA methyltransferases (DNMTs) and Ten-Eleven Translocation proteins (TETs). DNMTs generate and maintain the inheritance of 5-methylcytosine (5mC), which is the substrate targeted by the TET enzymes for conversion to 5-hydroxymethylcytosine (5hmC) and its downstream oxidized derivatives. The activity of DNMT and TET is dependent on the availability of micronutrients and metabolite co-factors, including essential vitamins, amino acids, and trace metals, highlighting how DNA methylation levels can be directly enhanced, suppressed, or remodeled via metabolic and nutritional perturbations. Dynamic changes in DNA methylation are required during embryonic development, lineage specification, and maintenance of somatic cell function that can be fine-tuned based on the influence of essential micronutrients. As we age, DNA methylation and hydroxymethylation levels drift in patterning, leading to epigenetic dysregulation and genomic instability that underlies the formation and progression of multiple diseases including cancer. Understanding how DNA methylation can be regulated by micronutrients will have important implications for the maintenance of normal tissue function upon aging, and in the prevention and treatment of diseases for improved health and lifespan.

## Introduction

DNA methylation in the form of 5-methylcytosine (5mC) can be found in ∼80% of CpGs in the genome and is primarily enriched in heterochromatin, working in positive feedback with silencing histone marks to maintain lineage identity (Lister et al., 2009). 5mC is conventionally known as a repressive epigenetic mark, controlling gene expression and chromatin organization to influence cellular development and differentiation (Guo et al., 2014). During development and in response to environmental stimuli, many 5mC marks are dynamic, including at shores of CpG islands, promoters, enhancers, and across gene bodies of differentially expressed genes (Deaton and Bird, 2011; Nguyen et al., 2022). DNA methyltransferases (DNMTs) catalyze the transfer of a methyl group to the 5’ position of cytosine residues to regulate *de novo* 5mC formation (DNMT3A and DNMT3B) independently of the cell cycle, or during DNA replication to ensure faithful DNA methylation inheritance (DNMT1) (Okano et al., 1999; Pradhan et al., 1999).

The Ten-eleven translocation (TET1-3) enzymes are considered to have an opposing role to the DNMTs due to their ability to oxidize 5mC to generate 5-hydroxymethylcytosine (5hmC), 5-formylcytosine (5fC), and 5-carboxylcytosine (5caC) (Tahiliani et al., 2009; Ito et al., 2010; He et al., 2011). TET proteins belong to a superfamily of dioxygenases that utilize Fe^2+^ and α-ketoglutarate (αKG) as essential cofactors (Lorsbach et al., 2003) [reviewed in (Lu et al., 2015a; Rasmussen and Helin, 2016; Wu and Zhang, 2017; Lio et al., 2020)]. The oxidized methylcytosines generated by TETs can be stable modifications in the genome or transient modifications that promote active or passive DNA demethylation (Tahiliani et al., 2009; Zhang et al., 2010; Bachman et al., 2014). DNMT1 preferentially recognizes hemi-methylated DNA to establish inheritance of methylation at palindromic CpG dinucleotides upon DNA replication (Bashtrykov et al., 2012) however, the inability of DNMT1 to recognize 5hmC causes a passive, cell-cycle dependent loss of DNA methylation (Otani et al., 2013). Unlike 5hmC that does not interfere with RNA or DNA polymerase extension, the iterative oxidation products 5fC and 5caC can slow RNA elongation (Wang L. et al., 2015), cause DNA polymerase pausing (Shibutani et al., 2014), and mimic a T:G mismatch leading to active removal and replacement with an unmethylated cytosine via base excision repair (BER) independently of DNA replication (Cortellino et al., 2011; He et al., 2011).

Essential micronutrients can directly or indirectly influence DNMT and/or TET activity that in turn impact DNA methylation levels (Figure 1). B vitamins can be regarded as pro-methylating micronutrients given their role in one-carbon metabolism that generates both nucleotides for DNA synthesis and the transfer of methyl groups required for the maintenance of DNA methylation (Tong et al., 2009; Lyon et al., 2020). The methyl group used in the formation of 5mC by DNMTs is derived from the coordinated effort of B2, B6, B9, B12, and the essential amino acid methionine. Together, these micronutrients regulate the folate and methionine cycles of one-carbon metabolism to generate S-adenosylmethionine (SAM), the sole methyl donor used by all methyltransferases to methylate proteins, RNA and DNA. TET catalytic activity is also dependent on metabolites and micronutrients whose abundance can be directly regulated by dietary supplementation. More recently it has been shown that vitamin C can act as a direct cofactor of TET enzymes to enhance 5hmC formation (Cimmino et al., 2017; Cimmino et al., 2018) by reducing ferric iron (Fe^3+^) to ferrous iron (Fe^2+^). Alterations in iron homeostasis may influence TET activity, as shown in the brain, liver, gut, lymphocytes, and cord blood cells (Jiang et al., 2021; Barks et al., 2022; Gao et al., 2022; Taeubert et al., 2022) and additional micronutrients, such as vitamin A, D, and E, have also been implicated as direct or indirect regulators of DNA methylation (Doig et al., 2013; Hore et al., 2016; Remely et al., 2017). Given that aberrant DNA methylation profiles are associated with aging (Sedivy et al., 2008; Salameh et al., 2020; He et al., 2021; Wilkinson et al., 2021; Lu et al., 2023) and that loss of function mutations in DNMT and TET enzymes are a hallmark of cancer (Delhommeau et al., 2009; Figueroa et al., 2010a; Li et al., 2011; Buscarlet et al., 2017; Ostrander et al., 2020), understanding the connection between DNA methylation maintenance and micronutrient availability may uncover liabilities associated with diseases upon aging (Patel et al., 2023) and reveal novel metabolic interventions that can be implemented to retain faithful DNA methylation patterns and function for disease prevention and longevity.

**FIGURE 1:**
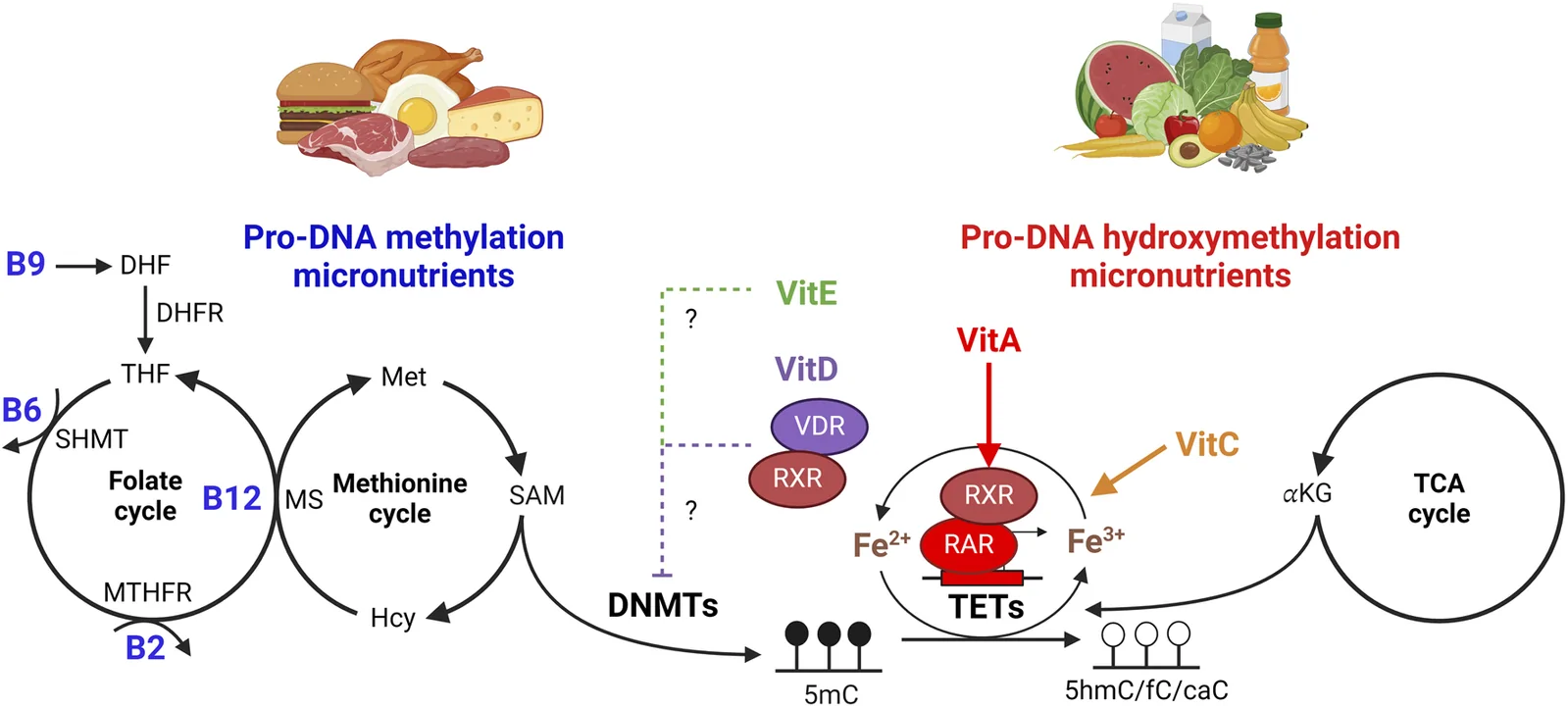
Role of micronutrients on DNA (hydroxy) methylation. One-carbon metabolism is comprised of the folate and methionine cycles that coordinate both nucleotide synthesis and the generation of S-adenosylmethionine (SAM), the sole methyl donor used by DNA methyltransferases to generate 5-methylcytosine (5mC) in the genome. Folate (vitamin B9) is converted to dihydrofolate (DHF) then to tetrahydrofolate (THF) by dihydrofolate reductase (DHFR). Serine hydroxymethyltransferase (SHMT) utilizes vitamin B6 as a cofactor to generate the metabolite 5,10-methyleneTHF, which can then be reduced by methylenetetrahydrofolate reductase (MTHFR) into 5-methyltetrahydrofolate (5-mTHF) using B2 as a cofactor. In conjunction with the methionine cycle, vitamin B12 is a cofactor for methionine synthase (MS), which uses 5-mTHF to transfer a methyl group to homocysteine (Hcy) in the recycling of methionine. Methionine, when converted to S-adenosylmethionine (SAM), provides DNA methyltransferases (DNMTs) with the methyl group needed to generate 5mC. Ten-Eleven Translocation (TET) enzymes hydroxylate 5mC to form 5hmC, 5fC, and/or 5caC using Fe^2+^ and α-ketoglutarate (αKG) as cofactors. Vitamin C (VitC) enhances TET function by recycling ferric (Fe^3+^) to ferrous (Fe^2+^) iron, while αKG is supplied by the tricarboxylic acid (TCA) cycle. Vitamin A (VitA) can directly upregulate TET gene expression via retinoid receptor (RAR/RXR) signaling. Vitamin D (VitD) acts as a ligand for the vitamin D receptor (VDR), which can also form a heterodimer with RXR, and both VitD or vitamin E (VitE) treatment have been shown to inhibit DNMT expression to influence DNA methylation maintenance. Figure created with BioRender.com.

## Micronutrient regulation of 5-methylcytosine formation and maintenance (pro-methylating micronutrients)

The role of several B vitamins in the direct regulation of one-carbon metabolism places these essential micronutrients as DNA methylation guardians of the epigenome. One-carbon metabolism is comprised of the folate and methionine cycles that coordinate both pyrimidine synthesis and the generation of S-adenosylmethionine (SAM), the sole methyl donor used by DNA methyltransferases to generate 5mC in the genome. The coordinated regulation of nucleotide synthesis and methylation by one-carbon metabolism ensures that DNA replication is coupled with DNA methylation inheritance (Ducker and Rabinowitz, 2017). A connection between the folate cycle and DNA methylation maintenance has been known for decades. Nutritional vitamin B9 or B12 deficiencies during gestation or polymorphisms in folate cycle enzymes of pregnant women are associated with neural tube defects (NTDs) (Smithells et al., 1976; Prevention of neural tube defects, 1991; Crider et al., 2011). The process of neurulation in embryos requires increased global methylation to promote neural tube fusion (Greene et al., 2011), and NTDs potentially form due to the lack of silencing of *Notch1* and *Sox2* (Alata Jimenez and Strobl-Mazzulla, 2022). Loss of serine hydroxymethyltransferase 1 (SHMT1) activity, which utilizes B6 as a cofactor, can impair neural tube closure (Beaudin et al., 2011; Beaudin et al., 2012). Moreover, polymorphisms that decrease the activity of methylenetetrahydrofolate reductase (MTHFR) (Prevention of neural tube defects, 1991), which depends on B2 as a cofactor, or methionine synthase (MS) that utilizes B12 as a cofactor (Tong et al., 2009; Guéant et al., 2020), and methionine synthase reductase that regenerates methylated B12 levels from its oxidized form have all been associated with NTDs (Botto and Yang, 2000; Imbard et al., 2013). One-carbon metabolism inhibitors such as the anti-folate methotrexate (MTX) that blocks the activity of dihydrofolate reductase (DHFR) promotes DNA hypomethylation in embryonic tissues and leads to NTDs (Greene et al., 2011; Wang X. et al., 2015). DNA methylation generated by DNMTs is required for closure of the neural tube, therefore as per one-carbon metabolic enzyme gene polymorphisms and B vitamin deficiencies, disruption of DNA methylation caused by *Dnmt3b* knockout, and DNMT or methylation cycle inhibitors also result in NTDs (Okano et al., 1999; Afman et al., 2005; Dunlevy et al., 2006).

The role of B vitamins in DNA methylation maintenance in other diseases and cell types have also been described. Clinical studies have shown that lower B9 intake leads to DNA hypomethylation in colonic tissue (Pufulete et al., 2003) and an increased risk of colorectal cancer (CRC) (Kim, 2007). A B9-deficient diet in post-menopausal women also causes DNA hypomethylation in lymphocytes (Jacob et al., 1998). Low dose therapy with MTX used in the treatment of rheumatoid arthritis demethylates the *FOXP3* locus in developing regulatory T cells, resulting in their enhanced differentiation and suppression of inflammation (Cribbs et al., 2015; Rossetti et al., 2015). Furthermore, higher global DNA methylation in leukocytes is associated with decreased responsiveness to MTX in rheumatoid arthritis patients (Gosselt et al., 2019), indicating the importance of MTX’s hypomethylating activity.

Elevated levels of B9 or B12 supplementation are positively correlated with increased DNA methylation through increased methyl-donor availability. Combined B9 and B12 supplementation leads to increased DNA methylation in leukocytes (Kok DE. et al., 2015; Kok DEG. et al., 2015), and B12 supplementation alone increases DNA methylation in disease-free children (Yadav et al., 2018) and mouse models of depression and meningitis (de Queiroz et al., 2020; Trautmann et al., 2020). Supplementation with B9 increases sperm DNA methylation levels in a mouse model of the *MTHFR* polymorphism, and supplementation with B2 has also been shown to increase DNA methylation in peripheral leukocytes of healthy individuals or cardiovascular disease patients with an *MTHFR* polymorphism (Amenyah et al., 2020; Amenyah et al., 2021). Amongst the healthy population, there have been no reports of toxicity for excess B vitamin consumption; however, given that one-carbon metabolism promotes DNA methylation, caution may be warranted for high B vitamin supplementation in disease states driven by DNA hypermethylation phenotypes (Ehrlich, 2019).

## Hydroxymethylation formation and DNA methylation removal via micronutrient mediated activation of the TET enzymes (pro-hydroxymethylation micronutrients)

Epigenetic plasticity, including the ability to remodel DNA methylation patterns in the genome, is integral to maintaining stem cell potency, lineage specification, and gene regulation in response to changes in the environment. The TET enzymes (TET1-3) are the only known mammalian DNA demethylases that can trigger the passive or active removal of 5mC in the genome (Tahiliani et al., 2009; Ito et al., 2010; He et al., 2011). TET1 is most highly expressed in embryonic stem cells, primordial germ cells, and neural tissues, whereas TET2 and TET3 are most highly expressed in stem, progenitor, and other differentiated adult cell populations (Lu et al., 2015a; Rasmussen and Helin, 2016; Wu and Zhang, 2017; Lio et al., 2020). While the TET enzymes all exhibit a conserved core C-terminal catalytic domain, they diverge in their N-terminal structure, in which TET2 lacks the CXXC DNA-binding motif found in TET1 and TET3 and therefore relies on protein-binding partners for its recruitment to chromatin (Ko et al., 2013; Rampal et al., 2014). TET activity is primarily associated with gene activation, given the enrichment of 5hmC within enhancers and gene bodies of actively expressed genes (Ficz et al., 2011; Rasmussen and Helin, 2016). The role of TET activity is perceived to oppose the gene silencing role of DNMTs; however, 5mC is an essential requirement for the generation of 5hmC and the iterative oxidized products 5fC and 5caC, thus DNA methylation and hydroxymethylation, as mediated by DNMTs and TETs, must work together to form and remove cytosine methylation in the DNA (Zhang et al., 2016; Gu et al., 2018; Lopez-Moyado et al., 2019; Chao et al., 2022). In addition to the 5mC DNA substrate, TET enzymes require Fe^2+^ and αKG as obligatory cofactors for their catalytic activity (Tahiliani et al., 2009). Perturbations in Fe^2+^ homeostasis can influence TET activity, where iron sufficiency is associated with increased TET activity and maintenance of 5hmC formation in cord blood, hepatocytes, enterocytes, and splenocytes (Jiang et al., 2021; Taeubert et al., 2022) whereas deficiencies in iron lead to reduced activity in the cerebellum of rats evidenced by decreased 5hmC levels and neurodevelopmental defects (Barks et al., 2022). However, in settings of excess iron and iron overloading, in which homeostatic responses may block cellular uptake of redox active iron (Conrad et al., 1963), TET enzymatic activity can also become impaired and promote pathogenic T cell expansion and systemic lupus erythematosus pathogenesis (Gao et al., 2022). These findings suggest that at a physiological level, the role of iron homeostasis and the balance between iron stores or redox active labile iron on TET activity requires further clarification.

Based on its role as a cofactor of αKG-dependent dioxygenases (αKGDDs), vitamin C was tested as an enhancer of TET activity. Acting as a targeted antioxidant, vitamin C can directly bind the C-terminal catalytic domain of Tet enzymes, promoting the reduction and recycling of Fe^3+^ to Fe^2+^ (Yin et al., 2013; Cimmino et al., 2017). Vitamin C has been shown to increase the levels of 5hmC, 5fC, and 5caC anywhere from 4 to 20-fold in a variety of contexts, including ESCs, fibroblasts and leukemia cells, within as little as 24 h (Wang et al., 2011; Minor et al., 2013; Yin et al., 2013; Cimmino et al., 2017; Mingay et al., 2018; Brabson et al., 2021). Increased 5hmC formation and DNA demethylation upon vitamin C treatment leads to improved reprogramming efficiency during iPSC formation, and enhanced pluripotency of ESC cultures (Hore et al., 2016) by reducing methylation normally gained at CpG islands (CGIs) of pluripotency genes during blastocyst to epiblast transition (Blaschke et al., 2013; Hu et al., 2014). Tissue specific studies of vitamin C treatment in lung, breast, bladder, and kidney cultures have also reported similar increases in 5hmC generation and DNA demethylation (Ge et al., 2018; Sant et al., 2018).

Interestingly, an algal homolog of the TET enzymes uses vitamin C directly instead of αKG as the primary cofactor for the generation of glucosyl-methylation marks in the genome to regulate photosynthesis (Xue et al., 2019). Vitamin C is a structural homolog of αKG, but unlike this TCA cycle intermediate, is an essential micronutrient for humans and therefore imparts a dependence for enhancing TET activity in the context of nutritional availability (Myllyla et al., 1978; Majamaa et al., 1986; Yin et al., 2013). One requirement for increased TET activity may be in the regulation of immune responses. In regulatory T (Treg) cells, vitamin C promotes DNA demethylation of a *Foxp3* enhancer, promoting Treg differentiation and immunosuppressive function akin to the role of low-dose MTX in rheumatoid arthritis patients (Yue et al., 2021; Suga et al., 2023). Vitamin C also drives oxidized methylcytosine formation and DNA demethylation within the three enhancers of the *Prdm1* locus that are silenced in naive B cells but require activation for plasma cell differentiation (Qi et al., 2020). The effect of vitamin C on lymphocyte differentiation, including Tregs and plasma cells, has been shown to be both Tet2 and Tet3 dependent, which are the most highly expressed of the three TET enzymes in hematopoietic cells (An et al., 2015; Brabson et al., 2023). Furthermore, vitamin C increases Th1-type chemokines and PD-L1 gene expression in a TET2-dependent manner resulting in enhanced sensitivity to anti-PD-1/PD-L1 therapy (Xu et al., 2019).

Deficiencies in vitamin C mimic loss-of-function mutations in *TET2,* which is the most frequently mutated of the TET enzymes in cancer and primarily in blood cell malignancy causing impaired DNA de-methylation. Aberrant DNA hypermethylation and/or vitamin C deficiency have both been reported in patients with hematological malignancies (Figueroa et al., 2010a; Figueroa et al., 2010b). Oral supplementation with vitamin C can raise plasma levels in patients with hematological malignancies, leading to an overall increase in the 5hmC/5mC ratio in circulating white blood cells (Gillberg et al., 2019) and a reduction in the proportion of hypermethylated loci caused by *TET2* mutation (Taira et al., 2023). Normalization of low vitamin C serum levels via supplementation in murine models also leads to a restoration of 5hmC formation in hematopoietic cells that suppresses *Tet2*-deficient leukemia progression (Agathocleous et al., 2017). These studies provide proof of principle that altering vitamin C supplementation can have a powerful influence on the blood cell DNA methylome.

Recent studies have also implicated vitamin A as a regulator of TET activity and DNA demethylation. Vitamin A comprises multiple active metabolites termed retinoids that play an essential role in growth, development, and differentiation (Gudas, 2013). The active form of vitamin A, retinoic acid (RA), binds to retinoic acid and retinoid X receptors (RAR/RXR) in the nucleus that, in association with other transcriptional coactivators, localize to retinoic acid responsive elements (RAREs) in target genes to activate gene expression (Gudas, 2013; Hore et al., 2016; Coyle et al., 2018). Thymidine DNA glycosylase (TDG) promotes DNA demethylation by forming a complex with acetylated TET2 (Zhang et al., 2017), and RAR/RXR activation upon RA-signaling has been shown to recruit the histone acetyltransferase CBP, TET proteins, and TDG to trigger oxidized methylcytosine formation and DNA demethylation via BER at target gene loci (Leger et al., 2014; Hassan et al., 2017; Hassan et al., 2020). RA-signaling in this manner can initiate TET2-dependent DNA demethylation at the *Hic1* locus, which is often hypermethylated in human cancers, such as colon, breast, and brain cancer (Hassan et al., 2020). RA-signaling in breast cancer cells also induces a RARB-TET2 complex, targeted to the promoters of genes involved in cellular differentiation, such as *miR-200c* (Wu et al., 2017). Defective RARB/TET2 signaling by loss of TET2 and/or deficient *miR-200c* expression is correlated with RA-resistant breast cancer cell growth and aggressiveness (Wu et al., 2017). RA treatment of human embryonic carcinoma stem cells has also described a TET2-dependent 5mC to 5hmC conversion in the *HOXA* gene cluster (Bocker et al., 2012). These studies collectively show that RAR, CBP, TET, and TDG enzymes play an interconnected role in the initiation of gene expression in response to RA-signaling. TET expression can also be directly up-regulated by RA-signaling, and alone or in combination with vitamin C, enhances 5hmC formation in ESCs (Hore et al., 2016). A conserved RARE identified in the first intron of the *TET2* locus suggests that expression could be mediated via direct RAR binding (Hore et al., 2016). Thus, the ability to directly enhance TET expression and increase the catalytic rate of TET activity via combined treatment with vitamin A and vitamin C suggests that these micronutrients work together to activate gene expression via increased methylcytosine oxidation.

## Indirect micronutrient regulation of DNA methylation

Other essential micronutrients have been reported to play an indirect role in the maintenance of DNA methylation via modulation of chromatin states and the regulation of DNA methyltransferase expression. Similar to RAR, the vitamin D receptor (VDR) forms a heterodimer with RXR at vitamin D responsive elements (VDREs) in the DNA (Doig et al., 2013; Krstic et al., 2022; Mirza et al., 2022). The active form of vitamin D, calcitriol [1,25(OH)_2_D_3_], when bound to VDR-RXR, triggers the recruitment of co-activators, including steroid receptor and p300/CBP (Fetahu et al., 2014). However, in the absence of its ligand, VDR-RXR is bound by co-repressors such as NCOR1 and SMRT that maintain repressive histone modifications at target loci (Doig et al., 2013; Fetahu et al., 2014). Vitamin D signaling may promote DNA hypomethylation via the downregulated expression of *DNMT1* and *DNMT3B* (Jiao et al., 2019; Lai et al., 2020) in this matter. Likewise, vitamin E can influence the expression of *DNMT1;* both a decreased expression in liver cells and an increased level in colon cells have been reported (Remely et al., 2017; Zappe et al., 2018). Prostate cancer models treated with vitamin E exhibit decreased DNMT protein expression and reduced CpG methylation at target loci of genes involved in cellular redox homeostasis such as the *Nrf2* promoter (Huang et al., 2012a). As oxidative stress is often correlated with increased global levels of 5mC (Garcia-Guede et al., 2020; Goncalves et al., 2021), it is possible that vitamin E can reduce DNA methylation indirectly due to its antioxidant activity (Ryan et al., 2010; Zappe et al., 2018).

## Discussion and future directions

The balance of essential micronutrient intake and bioavailability has the potential to significantly alter DNA methylation and hydroxymethylation levels in the body (Figure 2). Research using datasets comprising thousands of samples to track alterations in DNA methylation associated with cellular development, differentiation, aging, and disease progression has led to the identification of DNA methylation biomarkers and epigenetic clocks that accurately estimate chronological age (Hannum et al., 2013; Horvath, 2013; Horvath and Raj, 2018), healthspan and lifespan (Levine et al., 2018; Lu et al., 2019; Lu et al., 2022), or the pace of aging (Belsky et al., 2022) in both humans and animal models. These same DNA methylation signatures are also being used to investigate the impact of lifestyle factors such as diet and nutritional status on disease risk and aging (Quach et al., 2017; Lu et al., 2019; Fitzgerald et al., 2021) that may reveal potential therapeutic interventions to slow aging phenotypes and age-related diseases, including cancer. Plant-based, Mediterranean, and methylation-supportive diets can rejuvenate DNA methylation aging signatures, decreasing chronological age predictions by 1–10 years in as little as 8 weeks of intervention (Gensous et al., 2020; Dwaraka et al., 2023; Fitzgerald et al., 2023). One study (Dwaraka et al., 2023) using blood samples from paired twins reported that a vegan diet compared to an omnivorous diet led to a significant decrease in overall epigenetic age acceleration measured using multiple DNA methylation biomarkers (Levine et al., 2018; Lu et al., 2019; Belsky et al., 2022; Lu et al., 2022) that correlated with fewer hypermethylated loci in the vegan diet, consistent with the notion that vegan diets contribute to a reduced intake and lower serum levels of B12 (Gilsing et al., 2010; Niklewicz et al., 2023) and methionine (McCarty et al., 2009; Sanderson et al., 2019; Allen and Locasale, 2021), two of the major regulators of methyl-donor (SAM) levels. However, no controlled studies have attempted to address how supplementation with specific micronutrients associates with epigenetic clocks and DNA methylation biomarkers of aging or disease.

**FIGURE 2:**
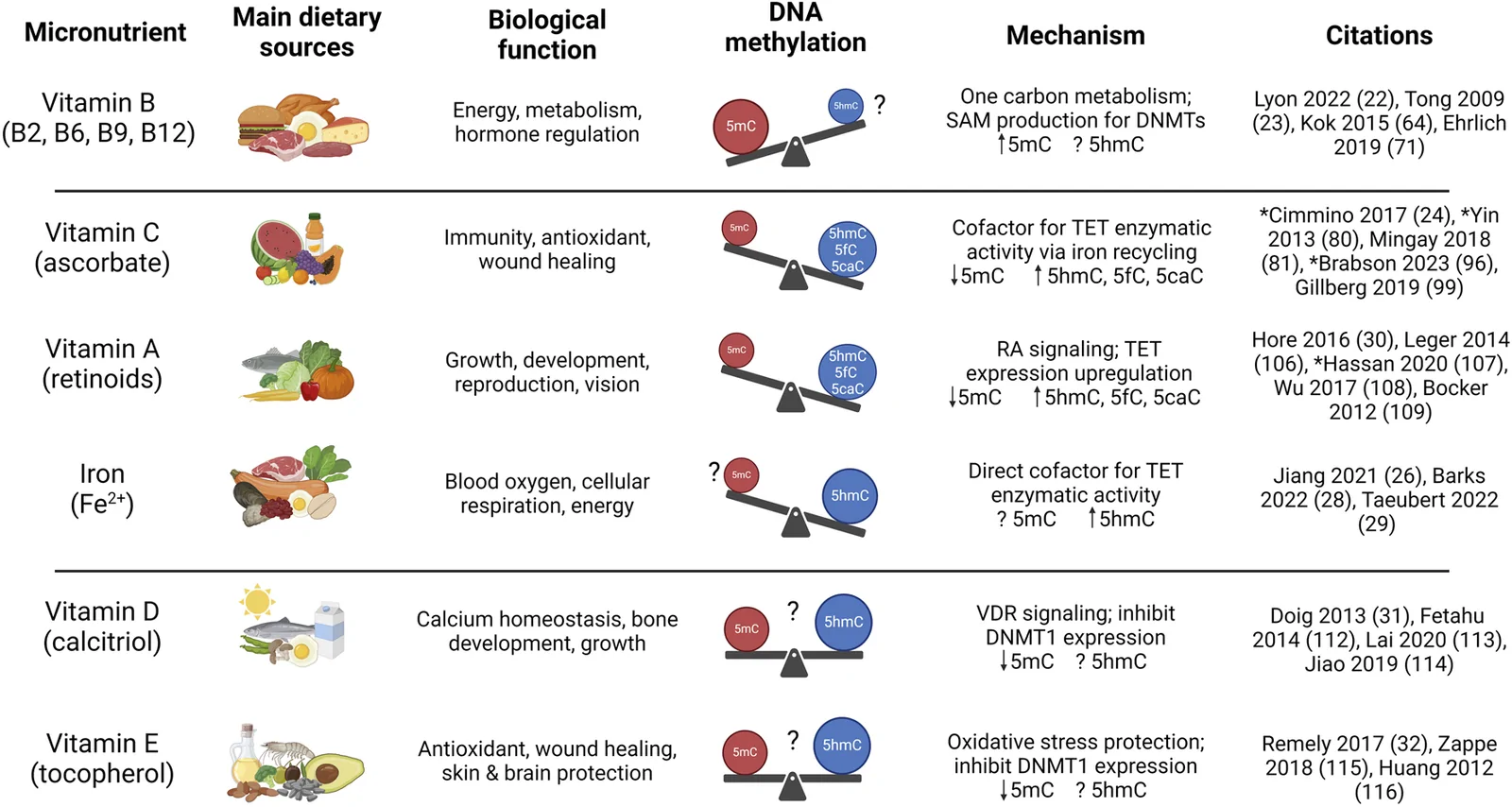
Influence of essential dietary micronutrients on DNA methylation or hydroxymethylation and biological outcome. Summary of biological effects of known micronutrient regulators of DNA methylation and hydroxymethylation. Vitamin A, typically sourced from foods such as carrots, leafy greens, and fish, is essential for development and reproduction, and stimulates TET activity leading to increased 5hmC formation and DNA demethylation. The various B vitamins play a coordinated role in energy production and one-carbon metabolism, are enriched in animal-derived foods, and higher intake can increase SAM production that facilitates DNMT activity and 5mC formation. Vitamin C, a major cellular antioxidant enriched in fruits and vegetables, acts as a cofactor for TET enzymes, driving 5hmC formation and DNA demethylation. Vitamin D is sourced from exposure to sunlight and is enriched in dairy products, whereas vitamin E, sourced from oils and nuts, protects against oxidative stress. Both Vitamin D and E have been shown to inhibit DNMT expression, although the effect on global methylation and hydroxymethylation varies based on cellular context and the mechanism remains unclear. Iron, a trace metal found in legumes, lean meat, and shellfish, is a direct cofactor for TET enzymes and increased supplementation facilitates 5hmC formation. *Studies that quantified micronutrient influence on 5hmC, 5fC, and 5caC levels. Figure created with BioRender.com.

An important caveat to consider when interpreting micronutrient influences on DNA methylation status using existing biomarkers is that the gold standard for measuring global DNA methylation in research and clinical diagnostics has historically relied on CpG hybridization array platforms using bisulfite-treated DNA. Unmethylated cytosines, 5fC, and 5caC, are converted to uracil upon bisulfite treatment, while both 5mC and 5hmC remain protected and measured as cytosine, rendering their contribution to DNA methylation signatures indistinguishable (Huang et al., 2010). DNA immunoprecipitation and base-resolution mapping of 5hmC, 5fC and 5caC in the mammalian genome have shown that most reside in a CpG context, enriched within gene bodies, low density CpG regions (CpG island shores), and at distal regulatory elements such as enhancers, where 5mC and 5hmC can be present in nearly equal representation (Williams et al., 2011; Wu et al., 2011; Huang et al., 2012b; Yu et al., 2012; Lu et al., 2015b; Xia et al., 2015). While the contribution of 5hmC, or the rarer modifications 5fC and 5caC, to the major DNA methylation biomarkers currently remains unknown, recent advances in base resolution mapping using alternate chemical and enzymatic conversion strategies (Huang et al., 2010; Booth et al., 2013; Schutsky et al., 2018) [reviewed in (Berney and McGouran, 2018)] should allow the contribution of nutritional interventions and micronutrient levels on DNMT vs. TET-mediated DNA methylation maintenance, epigenetic clocks, and other predictive disease biomarkers to be determined.
